# Convolutional neural networks for approximating electrical and thermal conductivities of Cu-CNT composites

**DOI:** 10.1038/s41598-022-16867-z

**Published:** 2022-08-10

**Authors:** Faizan Ejaz, Leslie K. Hwang, Jangyup Son, Jin-Sang Kim, Dong Su Lee, Beomjin Kwon

**Affiliations:** 1 School for Engineering of Matter, Transport and Energy, Arizona State University, Tempe, AZ 85287 USA; 2 Independent researcher, Chandler, Arizona USA; 3 Functional Composite Materials Research Center, Korea Institute of Science and Technology (KIST), Jeonbuk, 55324 Republic of Korea; 4 Division of Nano and Information Technology, KIST School University of Science and Technology (UST), Jeonbuk, 55324 Republic of Korea; 5 Institute of Advanced Composite Materials, Korea Institute of Science and Technology, Jeonbuk, 55324 Republic of Korea

**Keywords:** Energy science and technology, Materials science

## Abstract

This article explores the deep learning approach towards approximating the effective electrical and thermal conductivities of copper (Cu)-carbon nanotube (CNT) composites with CNTs aligned to the field direction. Convolutional neural networks (CNN) are trained to map the two-dimensional images of stochastic Cu-CNT networks to corresponding conductivities. The CNN model learns to estimate the Cu-CNT composite conductivities for various CNT volume fractions, interfacial electrical resistances, *R*_*c*_ = 20 Ω–20 kΩ, and interfacial thermal resistances, *R*^*″*^_*t,c*_ = 10^−10^–10^−7^ m^2^K/W. For training the CNNs, the hyperparameters such as learning rate, minibatch size, and hidden layer neurons are optimized. Without iteratively solving the physical governing equations, the trained CNN model approximates the electrical and thermal conductivities within a second with the coefficient of determination (*R*^*2*^) greater than 98%, which may take longer than 100 min for a convectional numerical simulation. This work demonstrates the potential of the deep learning surrogate model for the complex transport processes in composite materials.

## Introduction

Copper (Cu) is by far the most widely used conductive material in electronics, aviation, construction and power transmission lines. The progressive miniaturization and sophistication of high-power density devices demand copper alternatives to facilitate efficient electrical and thermal transport. Cu-carbon nanotube (CNT) composites are theoretically estimated to be superior electrical and thermal conductors to Cu at room temperature (27 °C)^1^. The conductivities of Cu-CNT composites are strongly influenced by CNT morphologies, i.e., CNT volume fraction and interfacial resistance at Cu-CNT and CNT-CNT interfaces. The electrical and thermal transport in CNT composites manifests in complex physics, which is extremely challenging to represent with closed-form models. Existing physics-based models, e.g., finite element model (FEM), are highly compute intensive, predominantly due to the extremely fine mesh required for CNTs and CNT-CNT interfaces.

Deep learning, a class of machine learning (ML), is applied in various scientific research areas to readily discover features from high-dimensional unstructured data (e.g., images, audio clips)^2,3^. For some nonlinear transport problems accompanied with complex physics in composites, deep learning algorithms interpret nonlinear patterns of data to classify or predict outputs without iteratively calculating the governing physical equations; thus, demanding lower computational costs than numerical simulation techniques^4–8^. Thus, researchers are actively investigating data driven deep learning analysis as an alternate modeling approach in composites^9–12^.

With the recent rapid development of ML methods, there has been growing interest in predicting the nanocomposite attributes without performing compute-intensive simulations. A previous study used convolutional neural networks (CNN) to predict thermal conductivity in composite materials^9^. 1500 composite material structures with volume fractions up to 30% were generated using the quartet structure generation set and effective thermal conductivities were calculated using the lattice Boltzmann method (LBM). The predicted results using CNN were found close to LBM with root mean square error (RMSE) of 1.9%. A past study utilized artificial neural networks (ANN) to determine the most favorable bridging alloying atom in Aluminum-CNT composite^10^. ANN was trained with 357 examples from literature for various alloying elements along with their strengthening efficiencies. The strengthening efficiencies approximated by the ML model were comparable to those of experiments with accuracy greater than 90%. Another research used ANN to predict the multiaxial strain-sensing response of CNT-polymer composites^11^. The ML model employed physics-based FEM at microscale to generate 15,000 examples to train ANN and approximated the macro-scale strain responses in CNT-polymer composites with accuracy of 99.65%. One previous record developed and trained the Gaussian Process Regression (GPR) model to predict the tensile strength in CNT-polymers nanocomposites^12^. The training data was collected from the available literature with 23 different polymers, combined with 22 CNT incorporating methods and 20 CNT modifications. The GPR model exhibited strong performance in predicting the tensile strength of CNT-polymer composites with training and validation accuracy of greater than 91%.

In this article, a convolutional neural network (CNN) is presented that infers the electrical and thermal conductivity of Cu-CNT composites at room temperature (27 °C) when an input data describing the stochastic distribution of CNTs, CNT volume fraction and Cu-CNT interfacial resistance is provided. The CNN model learns the important features from the images of Cu-CNT networks to predict the conductivities. To improve the accuracy of the CNN model, the influence of various hyperparameters such as learning rate, batch size and number of neurons in hidden layers is investigated. The trained CNN can serve as a surrogate model for Cu-CNT composite systems if the morphology of CNT network can be expressed in two-dimensional (2D) image format. For example, if the 2D images of Cu-CNT composites that sharply visualize the boundaries of CNTs, obtained either from computational modeling or processed microscopic images, are available, the trained CNN can rapidly examine the composite properties before conducting the expensive FEM or actual measurements.

## Training data generation

**Figure 1:**
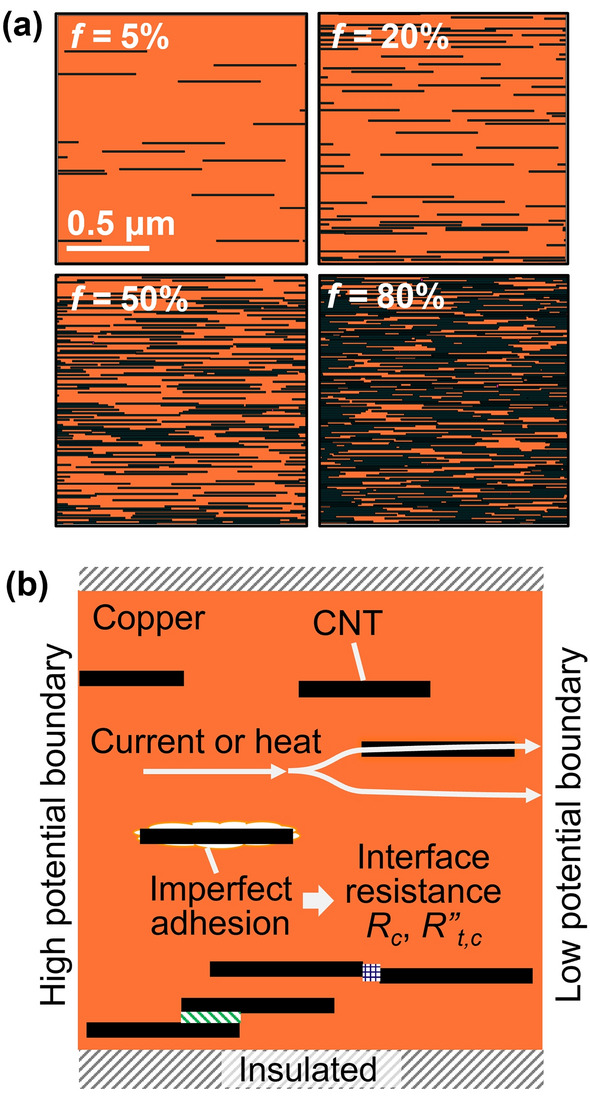
(**a**) Schematics of Cu-CNT networks with various CNT fractions *f*, (**b**) Schematic of a Cu-CNT network with boundary conditions. The patterned bars represent the side-to-side and end-to-end CNT-CNT interfacial resistance.

Training data is generated by creating the 2D stochastic Cu-CNT networks and simulating their electrical and thermal conductivities. A 2D finite element model (FEM) is used for the simulation that accounts for the CNT volume fractions, *f*, Cu-CNT interfacial resistances, and CNT-CNT interfacial resistances arising from the van der Waals interaction between two closely spaced CNTs. Since full details of FEM are available elsewhere^13^, only a minimal description follows. The 2D FEM model employs a simplified CNT morphology, i.e., straight CNTs aligned to the field direction, enabling the simulations of CNT networks with high volume fractions (up to 80%) at reduced computational costs. Several studies have reported that aligned, straightened CNTs exhibit enhanced electrical and thermal conductivities than entangled, randomly oriented CNTs^14–18^. Figure 1a illustrates some examples of Cu–CNT network models with various *f*. The 2D composite consists of non–overlapping CNTs (length 500 nm and width 10 nm) which are randomly distributed in the Cu matrix. Figure 1b shows the electrical and thermal boundary conditions used in FEM, which represent the following configurations: (1) steady-state electrical conduction and (2) heat conduction without internal heat generation. For electrical analysis, a potential difference, Δ*V*, of 1 μV is applied across the domain of length, *L*. For thermal analysis, the initial domain temperature is set to 27 °C and the temperature difference across the domain, Δ*T*, is kept at 1 °C. At Cu-CNT interfaces, the interfacial electrical resistance (*R*_*c*_) and interfacial thermal resistance (*R*^*″*^_*t,c*_) are defined in the ranges of *R*_*c*_ = 20 Ω–20 kΩ and *R*^*″*^_*t,c*_ = 10^−10^–10^−7^ m^2^K/W. The FEM estimates the electrical potential and temperature distributions in the Cu-CNT composite that are needed for the computation of effective electrical conductivity (*σ*_*e*_) and thermal conductivity (*k*_*e*_). The conductivities are normalized by the Cu matrix electrical conductivity (*σ*_*Cu*_ = 0.58 × 10^8^ S/m)^13^and thermal conductivity (*k*_*Cu*_ = 401 W/mK)^13^ at room temperature.

**Figure 2:**
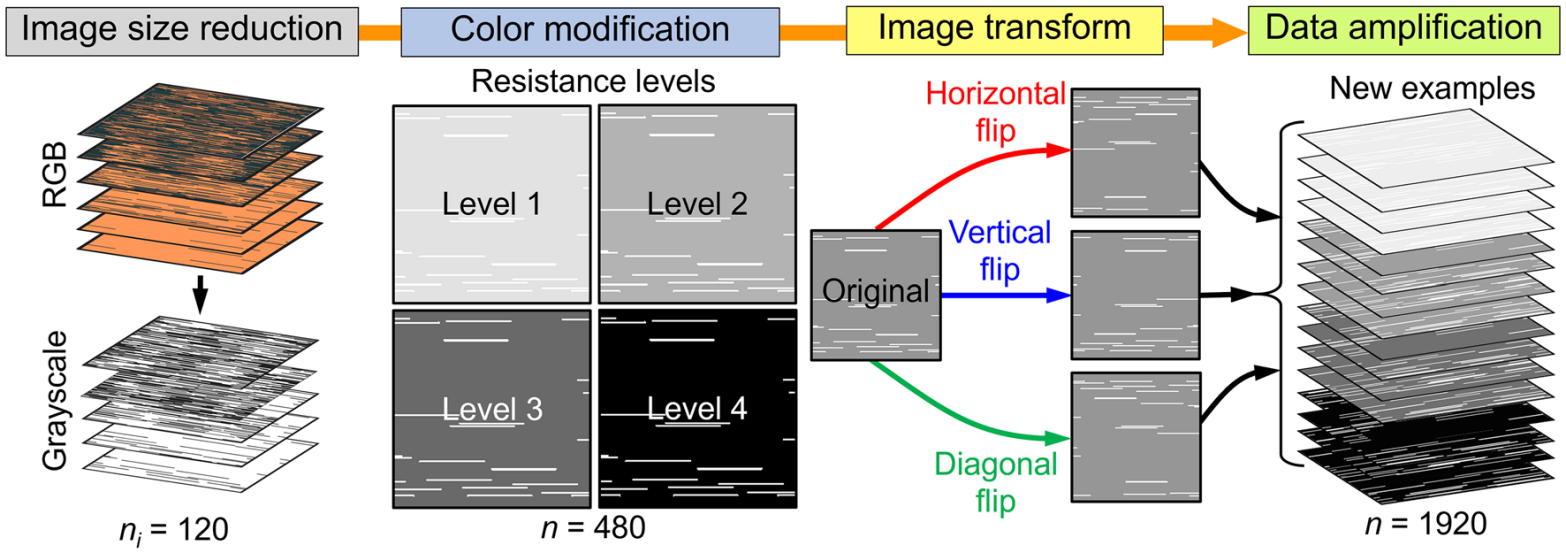
Schematic for the data preparation. The image data size of Cu-CNT networks is reduced by converting RGB scale into grayscale. The Cu-CNT interfacial resistance is encoded by the selection of Cu domain color intensity. The resistance levels are defined as follows. Level 1: *R*_*c*_ = 20 kΩ or *R*^*″*^_*t,c*_ = 10^−7^ m^2^K/W, Level 2: *R*_*c*_ = 2 kΩ or *R*^*″*^_*t,c*_ = 10^−8^ m^2^K/W, Level 3: *R*_*c*_ = 200 Ω or *R*^*″*^_*t,c*_ = 10^−9^ m^2^K/W, and Level 4: *R*_*c*_ = 20 Ω or *R*^*″*^_*t,c*_ = 10^−10^ m^2^K/W. The images are flipped horizontally, vertically, and diagonally to amplify the training dataset by four folds.

The training dataset is collected using FEM simulations and data augmentation. Figure 2 summarizes the data preparation process. First, 20 different images of Cu-CNT networks with random CNT distributions were generated for each target CNT fraction. Since 6 CNT volume fractions (i.e., *f* = 5%, 10%, 20%, 50%, 70% and 80%) were considered, in total, 120 Cu-CNT network images were created. Three-channel RGB images of Cu-CNT networks were converted into single-channel gray images to reduce the size of data. The information of Cu-CNT interfacial resistance was encoded in the Cu-CNT network image through a color code. The color intensity of the Cu domain was chosen by assigning grayscale intensities representing *R*_*c*_ or *R*^*″*^_*t,c*_, while the CNT regions were represented by white color (i.e., pixel intensity of 255). The pixel intensity of the Cu domain was varied as 0, 63, 129, 163 to encode four different levels of *R*_*c*_ and *R*^*″*^_*t,c*_. The total number of images after the color modification is increased to 480. The amount of training data was amplified using a simple image transformation technique, similar to a previous work^4^. As shown in Fig. 2, the original images were flipped in three ways: (1) horizontal, (2) vertical and (3) diagonal flips. The transformed Cu-CNT networks were assumed to possess identical conductivities to their original Cu-CNT network. With the data augmentation, the total number of Cu-CNT network models is increased to 1920. Finally, the Cu-CNT network images and tabulated electrical and thermal conductivities from FEM simulations were paired as the training dataset.

## Convolutional neural network

**Figure 3:**
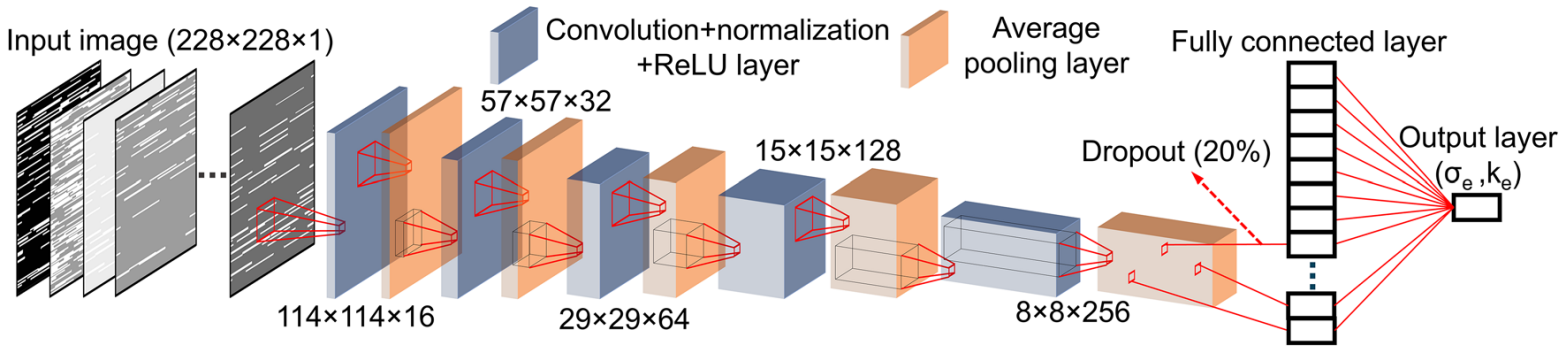
The architecture of CNN to approximate the effective electrical and thermal conductivities of Cu-CNT networks. *σ*_*e*_ denotes the effective electrical conductivity and *k*_*e*_ denotes the effective thermal conductivity.

Convolutional neural network (CNN) is a class of deep neural networks which is widely-used in image recognition tasks with remarkable success^19^. There are several CNN models with different structures successfully applied for image recognition such as AlexNet^20^, ResNet^21^, LeNet-5^22^, etc. The CNN model outperforms other machine learning algorithms in terms of non-linear function approximation and the ability to extract and articulate data features^23^. Thus, compared to conventional artificial neural networks such as multilayer perceptron and feed-forward networks, the CNN significantly reduces the computational demands when processing high-dimensional image information due to the feature parameter sharing and dimensionality reduction. Figure 3 shows the architecture of our CNN model obtained through hyperparameter tuning which is discussed in the next section. The CNN model consists of an input layer (i.e., Cu-CNT network), an output layer (i.e., predicted conductivities) and 6 hidden layers. The input layer is a single channel Cu-CNT network image, equivalent to a 228 × 228 × 1 matrix. The image size was chosen to retain high resolution and capture minuscule details of CNT networks, particularly at high CNT fractions. A convolution layer is added to generate feature maps from the input layer. The convolutional layer contains a series of 3 × 3 kernels which are convoluted with inputs to extract features while preserving the spatial relationships between image pixels. The batch normalization layer is added after every convolution layer to normalize and standardize the inputs between 0 and 1. A rectified linear unit activation (ReLU) layer is added to prevent the vanishing gradient problem, allowing the model to learn faster with improved stability. To down-sample the input feature map, a pooling layer with a filter size of 2 and stride of 2 is inserted after every activation layer. The pooling layer applies an average pooling operation in a prescribed filter size and abstracts the input feature maps, reducing the low-level features while extracting high-order features. After 6 iterations of hidden layers, a fully connected layer takes all the outputs in the previous layer and connects them to its single neuron, i.e., a one-dimensional feature vector. The feature vector represents the major features of the original input and can be used to establish the regression model for the electrical or thermal conductivities.

To train the CNN model, stochastic gradient descent (SGD) algorithm is used. SGD is one of the popular iterative optimization techniques for determining weights that minimize the errors in neural networks. SGD calculates the gradients on small randomized subsets of the training set, called minibatch. The gradient is calculated in small-steps called learning rate which determines the moving step size from one point to the next point with a negative gradient. After a full forward and backward pass on the complete training dataset, i.e., 1 epoch, the model weights are updated. By testing with a minibatch in the range of 5–20 and learning rate in the range of 10^−2^–10^−7^, we selected an optimal minibatch size as 20, a learning rate as 10^−3^ and epochs as 400. The learning rate was dropped by a factor of 0.1 after every 150 epochs, allowing the model to learn an optimal set of weights. The model training begins by initiating the kernel parameters using Gaussian initialization method which extracts the features of the Cu-CNT network. The kernel parameters are optimized according to the Euclidean loss function, $\left( {1/n} \right)\sum\nolimits_{i = 1}^{n} {\left\| {y_{i} - y_{{i^{\prime}}} } \right\|^{2} }$, which calculates the square sum of the difference between the two training outputs, i.e., predictive value, *y*_*i*_ and known value, *y*_*i*_*′*. The loss function is subsequently minimized after each iteration by updating the parameters.

## Results and discussion

The number of neurons in hidden layers was adjusted to balance the model accuracy and training time. The coefficient of determination (*R*^*2*^) was employed to quantitatively examine the model accuracy. Table 1 summarizes the *R*^*2*^ of training dataset (*R*^*2*^_*Train*_) and the validation dataset (*R*^*2*^_*Valid*_) along with model training time as a function of neurons in each convolution layer for both *σ*_*e*_*/σ*_*Cu*_ and *k*_*e*_*/k*_*Cu*_ predictions. The model training was performed on a graphic card (Nvidia RTX A6000) with 48 GB memory. In general, as the number of neurons, equivalently the depth of output volume, increases, both training and validation *R*^*2*^ increases along with the cost of additional training time. In our experiment, the number of neurons used in case 4 provided the highest *R*^*2*^_*Train*_ and *R*^*2*^_*Valid*_ (≥ 0.98) with a training time of ~ 3 min. The model *R*^*2*^ was not improved by further increasing the number of neurons as seen in case 5. Therefore, the number of neurons in each layer was chosen to be 16, 32, 64, 128 and 256 for all subsequent CNN training.

**Table 1:** CNN model *R*^*2*^ and training time obtained with various hidden layer neurons.

Case	Neurons in each layer	*σ*_*e*_*/σ*_*Cu*_ predictions			*k*_*e*_*/k*_*Cu*_ predictions		
		*R*^*2*^_*Train*_	*R*^*2*^_*Valid*_	Time (s)	*R*^*2*^_*Train*_	*R*^*2*^_*Valid*_	Time (s)
1	10, 20, 30, 40, 50, 60	0.83	0.79	110	0.83	0.71	114
2	20, 40, 60, 80, 100, 120	0.87	0.86	144	0.89	0.87	152
3	40, 80, 120, 160, 200, 240	0.96	0.91	280	0.94	0.90	299
4	8, 16, 32, 64, 128, 256	0.99	0.98	170	0.99	0.98	175
5	16, 32, 64, 128, 256, 512	0.99	0.94	352	0.98	0.92	322

**Figure 4:**
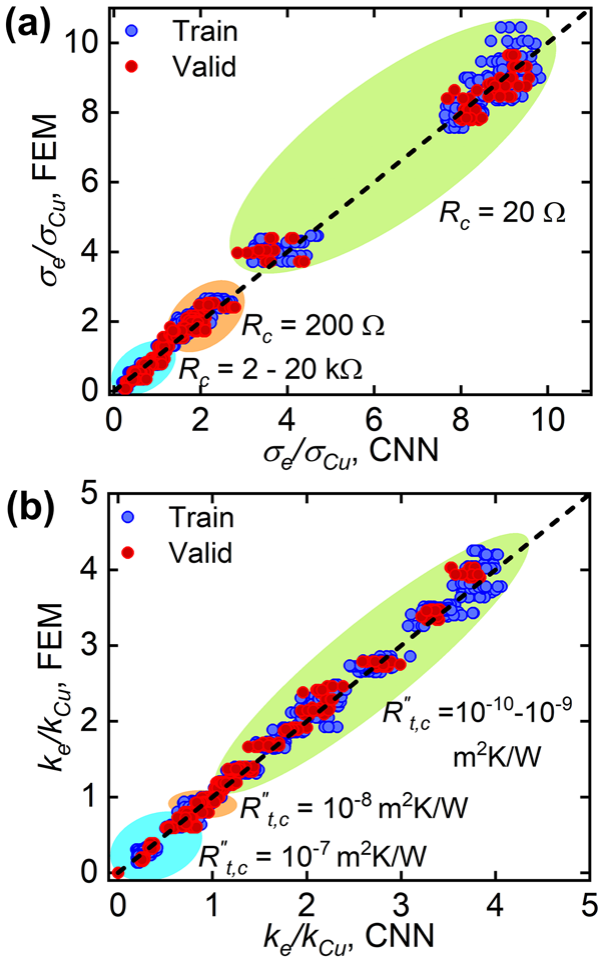
Comparison between CNN approximations and FEM predictions for (**a**) effective electrical conductivities with *R*^*2*^_*Train*_ = 0.991, *R*^*2*^_*Valid*_ = 0.982, and (**b**) effective thermal conductivities with *R*^*2*^_*Train*_ = 0.992, *R*^*2*^_*Valid*_ = 0.986.

The CNN was trained to predict the electrical and thermal conductivities of the Cu-CNT networks over wide range of interfacial resistances, i.e., *R*_*c*_ = 20 Ω–20 kΩ and *R*^*″*^_*t,c*_ = 10^−10^ m^2^K/W–10^−7^ m^2^K/W. Figure 4 compares the CNN model approximations and FEM predictions for *σ*_*e*_*/σ*_*Cu*_ and *k*_*e*_*/k*_*Cu*_. Overall, the training of CNN was successful with *R*^*2*^_*Train*_ ≥ 0.99, and the trained CNN was able to accurately predict the unseen Cu-CNT network models with *R*^*2*^_*Valid*_ ≥ 0.98. Note that training the CNN with 1920 Cu-CNT models took only ~ 3 min. With this training cost, the CNN model can estimate the conductivity of an unseen Cu-CNT network within 1 s, whereas the FEM requires ~ 155 min on average for the same task. Such characteristics of the CNN model suggest that the deep learning approach is a promising method when it is necessary to rapidly and repetitively estimate the properties of stochastic composite materials if the training dataset, *i.e.,* images of composite materials and corresponding properties, is available.

The training and validation datasets were designed to include diversified examples with various CNT fractions and interfacial resistances. The diversity in training data critically affects whether the neural network is able to overcome the bias or not. In our dataset, *σ*_*e*_*/σ*_*Cu*_ ranges from 0.08 to 10.45 and *k*_*e*_*/k*_*Cu*_ ranges from 0.15 to 4.25 as shown in Fig. 4. For the data generated with a large interfacial resistance (i.e., *R*_*c*_ = 20 kΩ and *R*^*″*^_*t,c*_ = 10^−7^ m^2^K/W), the Cu-CNT composites with high *f* (i.e., *f* ≥ 50%) possessed effective conductivities that were smaller than that of copper (i.e., 0 < *σ*_*e*_*/σ*_*Cu*_, *k*_*e*_*/k*_*Cu*_ < 0.5). For the examples with a large *R*_*c*_, *R*^*″*^_*t,c*_ and small *f* (i.e., *f* < 20%), the effective conductivities were close to unity. When the interfacial resistance is small (i.e., *R*_*c*_ = 20 Ω and *R*^*″*^_*t,c*_ = 10^−10^ m^2^K/W), the examples with high *f* (i.e., *f* ≥ 50%) exhibited effective conductivities that were greater than that of copper (i.e., 7.5 < *σ*_*e*_*/σ*_*Cu*_ < 11 and 2 < *k*_*e*_*/k*_*Cu*_ < 4.5). By combining various levels of *f*, *R*_*c*_ and *R*^*″*^_*t,c*_, the dataset incorporated the examples having effective conductivities similar to previously reported Cu-CNT composites^24–31^.

The method introduced in this article demonstrates that the deep neural networks can rapidly approximate the complex relation between the morphology of fiber composites and their electrical and thermal transport properties. The introduced approach will be useful for the researchers who need a surrogate model for fiber composite systems that estimates the composite properties before the expensive finite element simulations or actual measurements. Thus, the application of the introduced approach for inferring the properties of actual composite materials can be an extension of this work. Since the images of Cu-CNT composites used in this work showed the shapes of CNTs distinctly without any blurriness, the CNN readily recognized the layouts of CNTs and made predictions accurately. For the application of the introduced approach to actual materials, it will be necessary to acquire microscopic images of the samples from various parts and process the images to extract the morphology of CNT network similar to Fig. 1a while eliminating the background image features.

## Conclusions

This work reports a CNN that is trained to approximate the effective electrical and thermal conductivities of stochastic Cu-CNT networks when their 2D images are provided as inputs. The CNN architecture and hyperparameters were optimized to make approximations with *R*^*2*^ > 0.98. Despite the complex and nonlinear transport mechanism, the CNN predicted for unseen Cu-CNT networks of various CNT volume fractions and Cu-CNT interfacial resistances with the *R*^*2*^ greater than 98%. To provide a variety of learnable examples in CNN without performing additional FEM simulations, a simple image augmentation technique was used to diversify the training dataset by 4-folds. A possible extension of this work is to investigate the potential of CNN or other deep learning methods as rapid prediction models for microscopic images of fabricated bulk-scale Cu–CNT networks or other composite materials.

## Data Availability

The data included in this study is available from the corresponding author upon request as needed.
